# Supplementary figures and images for: Ramification has little impact on shoot hydraulic efficiency in the sexually dimorphic genus Leucadendron (Proteaceae)
Source: PeerJ. 2019 May 29;7:e6835. doi: 10.7717/peerj.6835 (PMC6545101; doi:10.7717/peerj.6835)

HP ramification

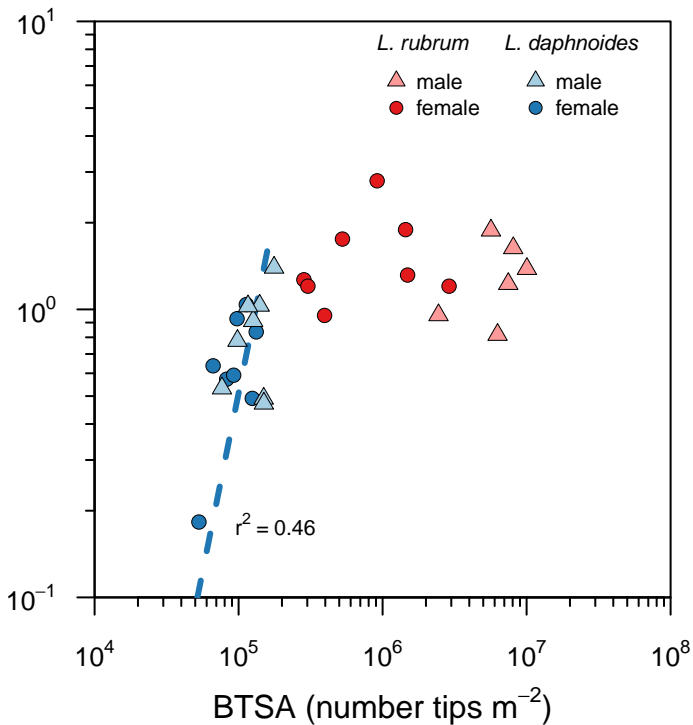

Supplement: Supplemental Information 1 — Relationship between the two metrics of ramification: that used by Harris & Pannell (2010); ‘HP ramification’) and that used in the present study (BTSA). Only within L. daphnoides was there a correlation between the two metrics of ramification. [file peerj-07-6835-s001.pdf]

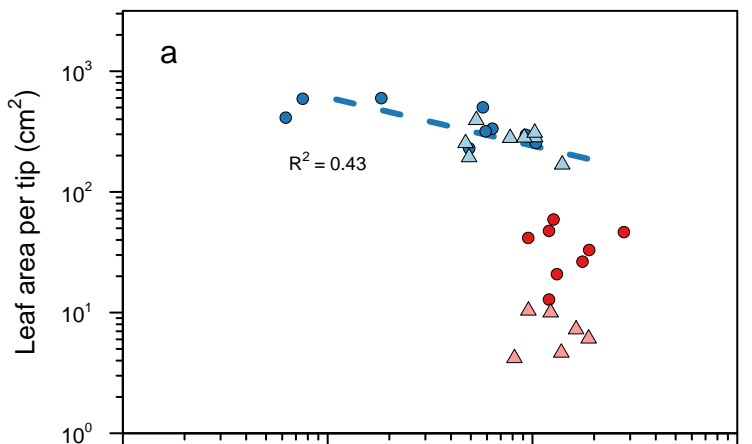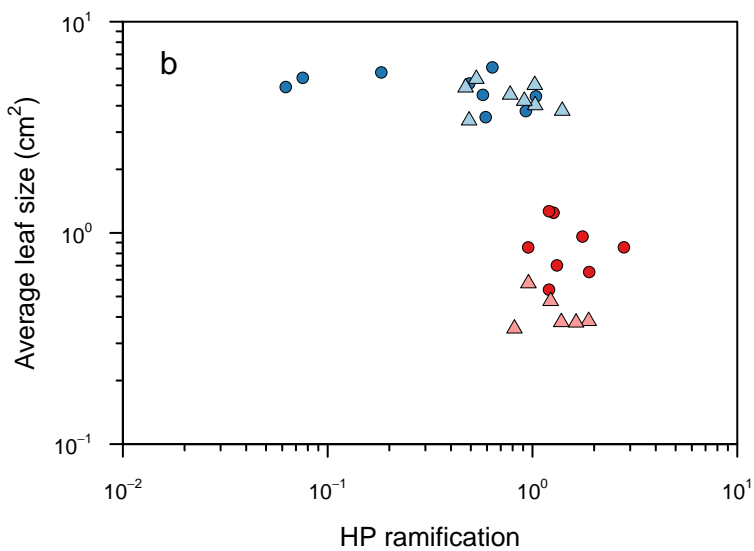

Supplement: Table S1 — Relationships between the metric of ramification used by Harris & Pannell (2010) (termed here ‘HP ramification’) and (a) the average leaf area per branch tip and (b) the average leaf size. There was a significant relationship between HP ramification and the leaf area per branch tip only for L. daphnoides (R2= 0.43, P = 0.05). No other relationships were significant, despite the expected scaling relationship between ramification and leaf size according to Corner’s rules (Corner, 1949). [file peerj-07-6835-s002.pdf]
